# Anti-roma Bias (Stereotypes, Prejudice, Behavioral Tendencies): A Network Approach Toward Attitude Strength

**DOI:** 10.3389/fpsyg.2020.02071

**Published:** 2020-09-30

**Authors:** Hadi Sam Nariman, Márton Hadarics, Anna Kende, Barbara Lášticová, Xenia Daniela Poslon, Miroslav Popper, Mihaela Boza, Andreea Ernst-Vintila, Constantina Badea, Yara Mahfud, Ashley O’Connor, Anca Minescu

**Affiliations:** ^1^Doctoral School of Psychology, Eötvös Loránd University, Budapest, Hungary; ^2^Institute of Psychology, Eötvös Loránd University, Budapest, Hungary; ^3^Department of Social Psychology, Eötvös Loránd University, Budapest, Hungary; ^4^Institute for Research in Social Communication, Slovak Academy of Sciences, Bratislava, Slovakia; ^5^Department of Psychology, Alexandru Ioan Cuza University of Iaşi, Iaşi, Romania; ^6^Université Paris Nanterre, Laboratoire Parisien de Psychologie Sociale, Nanterre, France; ^7^Department of Psychology, University of Limerick, Limerick, Ireland

**Keywords:** anti-roma bias, attitude strength, network connectivity, network analysis, intervention

## Abstract

The Roma have been and still are a target of prejudice, marginalization, and social exclusion across Europe, especially in East-Central European countries. This paper focuses on a set of stereotypical, emotional, and behavioral evaluative responses toward Roma people selected as representing the underlying components of anti-Roma bias. Employing network analysis, we investigated if attitude strength is associated with stronger connectivity in the networks of its constituent elements. The findings from representative surveys carried out in Hungary, Romania, Slovakia, France, and Ireland supported our assumption, as high attitude strength toward the Roma resulted in stronger connectivity in all pairs of high- versus low-attitude-strength networks. Our finding yields a solid theoretical framework for targeting the central variables—those with the strongest associations with other variables—as a potentially effective attitude change intervention strategy. Moreover, perceived threat to national identity, sympathy, and empathy were found to be the most central variables in the networks.

## Introduction

The Roma are among the most disenfranchised, socially unaccepted, and morally vilified ethnic minority groups in Europe and especially in East-Central European countries (Fraser, 1995; Ladányi, 2001; Pogány, 2006; Tileagã, 2006). As a culturally and linguistically diverse group, Roma people are portrayed as beggars, criminals, profiteers, and lazy, being a target of marginalization and social exclusion, as well as perpetual discriminatory and violent practices on an interpersonal, institutional, and national level (van Baar, 2011; Feischmidt et al., 2013). School segregation of Roma students in Hungary, the Czechia, and Slovakia (Messing, 2017), violent vigilante activities in Hungary and Romania, and forced eviction of the Roma in Romania, France, Italy, and Slovakia are all strikingly telling cases in point (see, e.g., Amnesty International Report, 2013).

Empirical research shows that anti-Roma stereotypes revolve around criminality, laziness, and receiving undeserved benefit from the state (e.g., Enyedi et al., 2004; Kende et al., 2017, 2020; Villano et al., 2017). Moreover, drawing on the stereotype content model (SCM, Fiske et al., 2002), the Roma are perceived to be low in both warmth and competence (e.g., Stanciu et al., 2017; Grigoryev et al., 2019). Further, research shows that the Roma are perceived as both dangerous and derogated (e.g., Imhoff and Bruder, 2014; Bilewicz et al., 2017; Hadarics and Kende, 2019), which also indirectly implies that they are both rejected from the perspective of threatening conventional norms and looked down upon as a low-status group—being low in both dimensions of the model.

Needless to say, intervention efforts are needed to combat anti-Roma bias. However, one practical challenge is to identify the most effective attitude change interventions considering that anti-Roma stereotypes are historically rooted and strong in most societies. Previous intervention efforts, in general, have not been successful in dampening intergroup bias (Paluck and Green, 2009). Mainstream intergroup bias research is often engaged with parsimonious models investigating relationships between a limited number of variables, which does not ensure identifying the most influential stereotypical and prejudicial evaluations. In the current study, we attempt to fill this gap by employing a network approach in the anti-Roma stereotype context. Our main objective is to examine whether the network approach would be a theoretically justified method to be employed for intervention purposes in an anti-Roma bias context in future research. Drawing on the literature on attitude strength and network analysis, we test the *connectivity hypothesis* proposed by Dalege et al. (2018) in the networks of stereotypical, emotional, and behavioral evaluations toward the Roma estimated from representative samples collected in Hungary, Romania, Slovakia, France, and Ireland.

The five countries included are the three Eastern European countries with the largest indigenous Roma minority (with 8% of the Romanian population, 7% in Hungary, and 9% in Slovakia) and two Western European countries (Ireland and France) where Roma have immigrated in the last 20 years and that also have their own indigenous Roma population groups (i.e., Irish Travelers in Ireland and Sinti in France). While their visible economic disadvantages may be the strongest in Eastern Europe, where they form a large (often the largest) ethnic minority group, their treatment in Western Europe is often inhumane and goes against EU norms and regulations (Mahoney, 2011; European Commission, 2015; Gould, 2015).

### Network Analysis

Network analysis is a relatively novel approach to modeling individual differences in psychological constructs by representing the direct interactions between their underlying components. Representing stereotype structures through network models has also recently received attention from researchers in the field (e.g., Sayans-Jiménez et al., 2018; Grigoryev et al., 2019). Modeling the direct and unique interrelations between a relatively higher number of variables as a network can be an advantageous method to render possible picturing of a more comprehensive representation of stereotype dynamics. Having a variety of stereotypes and negative attitudes estimated as a network can help us in finding variables with the highest degree of interrelations with other variables that can be the most favorable candidates to be wagered on for intervention purposes. With a latent approach, for instance, this cannot be possible, since all the items are treated as equivalent measures of the latent construct (Schmittmann et al., 2013).

Nodes and edges are the two most basic constituent elements of a network; nodes are the number of entities, and edges, the direct interrelationships between every possible pair of nodes. In psychological networks, nodes are a set of observed variables, and edges, the statistical associations between them (Epskamp et al., 2018). Connectivity is another basic property of a network that refers to the overall level of interrelations among all the nodes and the degree of causal interdependencies between them. The higher the connectivity between nodes within a given network, the more likely it is that changes to one node will also be mirrored by changes in other nodes within that network (Scheffer et al., 2012). Moreover, global connectivity, as a measurement of network connectivity, is the sum of all absolute values that every edge in the network possesses. Hence, the number of connections and the magnitude of the edge weights determine the connectivity of a network.

### Network Connectivity as Related to Attitude Strength

Proposing the Causal Attitude Network (CAN) model, Dalege et al. (2018) integrated the general notion of network connectivity with attitude networks and proposed the *connectivity hypothesis*, which refers to the higher connectivity between the evaluations on different aspects of an attitude object for those who hold a stronger attitude toward that attitude object.

As mentioned above, identifying the nodes with the highest degree of direct interactions with the other nodes in a network of stereotypical evaluations would be a highly beneficial means for intervention purposes. To consolidate this approach, in the current study, we employ the connectivity hypothesis. We argue that the connectivity between different stereotypical, emotional, and behavioral evaluations toward the Roma estimated as a network, to be found also as a measurement of attitude strength, would yield a firm theoretical linchpin for intervention aims. For if nodes with the highest interrelations with the others rendered at odds with the other nodes, the need for cognitive consistency as a factor indispensable to attitude strength (e.g., Simon et al., 2004; Monroe and Read, 2008) would lead the system to regain the compatibility between all its components.

By definition, attitude strength is “the extent to which attitudes manifest the qualities of durability and impactfulness” (Krosnick and Petty, 1995, p. 3). Durability refers to attitude stability over time and resistance to change, and impactfulness, to its influence on information processing and behavior. Strong attitudes, therefore, acquire these attributes to a greater extent in comparison with weak attitudes. Krosnick and Petty (1995) propose several features of attitude strength such as extremity, importance, and accessibility inter alia. Dalege et al. (2018) found that in a network of a number of evaluations on the presidential candidates, the network connectivity is higher for those who hold a stronger attitude concerning political campaigns. Moreover, they showed that network connectivity is also an expression of other basic properties of attitude strength. They estimated correlation coefficients between feeling thermometer items toward the presidential candidate measured before and after the election (as a measure of attitude stability) and found that network connectivity is significantly associated with attitude stability over time. Moreover, they also showed that network connectivity predicts the biserial correlations between the feeling thermometer item toward the presidential candidate before the election and the respondents’ actual voting decision (see Dalege et al., 2018).

In the current research, we test the connectivity hypothesis in the context of anti-Roma bias. In line with previous findings of the CAN model, we assume that high-attitude-strength networks of a number of stereotypical, emotional, and behavioral evaluations toward the Roma will possess a significantly stronger degree of global connectivity compared to those of low-attitude-strength networks.

## Method

Twenty-seven stereotypical, emotional, and behavioral evaluative responses toward the Roma (for an overview of the underlying components of an attitude, see McGuire, 1990) were used to examine their connectivity in the networks of high versus low attitude strength for each country. Four steps of network data analysis were performed: network estimation, network comparison, network inference, and network stability, recommended by Fried et al. (2018). Moreover, an additional check section was added to report the results of pathway analyses.

### Participants

Nationally representative survey data were collected through online participant pools across five countries; Hungary (*N* = 1,039, *M*_age_ = 47.99, SD_age_ = 14.84, 52.7% women), Romania (*N* = 1,044, *M*_age_ = 42.11, SD_age_ = 15.80, 48.2% women), Slovakia (*N* = 1,033, *M*_age_ = 44.06, SD_age_ = 16.10, 52.7% women), France (*N* = 975, *M*_age_ = 42.10, SD_age_ = 13.30, 54% women), and Ireland (*N* = 1,000, *M*_age_ = 44.91, SD_age_ = 15.72, 51.5% women).

Based on simulation studies (Epskamp, 2016), a moderate-size network with 24 nodes for continuous data is recommended to be estimated from at least 250 respondents approximately. The number of participants for all networks was sufficient (Hungary: *N*_high_ = 511, *N*_low_ = 512; Romania: *N*_high_ = 467, *N*_low_ = 463; Slovakia: *N*_high_ = 516, *N*_low_ = 517; France: *N*_high_ = 472, *N*_low_ = 498; Ireland: *N*_high_ = 476, *N*_low_ = 469). Moreover, 16 respondents from the Hungarian sample, 114 respondents from the Romanian sample, 5 respondents from the French sample, and 55 respondents from the Irish sample did not respond on the feeling thermometer scale and were removed from the analysis.

Data were collected by professional opinion poll companies in each country, working with the IRB approval of Eötvös Loránd University. The surveying companies used a multiple-step, proportionally stratified, probabilistic sampling method of an online participant pool, resulting in a sample demographically similar to the respective population in terms of age, gender, and type of settlement. Note that the French sample was representative only regarding age and gender. (See Supplementary Material for the demographic similarities between each sample and the corresponding population).

### Measures

Twenty-seven items of stereotypes, emotions, and collective action tendencies toward the Roma were selected for the network estimations from the omnibus surveys. A 14-item revised Attitudes Toward Roma Scale^1^ (original ATRS; Kende et al., 2017), with three subscales, was used. Six items of ATRS measured Blatant Stereotyping (e.g., “There are very little proper or reasonable Roma people.”), five items measured Undeserved Benefits (e.g., “The real damage is caused by organizations which offer an undeserved advantage to Roma people.”), and three items measured Cultural Difference (“The Roma can be proud of their cultural heritage.”). Four discreet intergroup emotions were measured, each with a single item: empathy (“I feel empathy with Roma people”), sympathy (“I feel sympathy with Roma people.”), anger (“I feel anger about the treatment of Roma people.”), and hope (“I feel hopeful about the future of Roma people.”). Collective action intentions with a pro-Roma orientation were measured by six items, including items on engagement in traditional forms of collective action, such as signing petitions [e.g., “I would participate in some form of action (e.g., signing a petition) defending the rights of the Roma.”] as well as items about offering donations and volunteerism (e.g., “I would donate clothing, school supplies or toys for Roma families.”). Lastly, three items measured perceived threat to national identity [e.g., “Roma people are a threat to (country) culture.”]. All the items were measured on a seven-point scale (1 = strongly disagree; 7 = strongly agree).

As a general measure of attitude, we used a single-item feeling thermometer scale measuring participants’ attitudes toward the Roma from 0 (very unlikeable) to 100 (very likeable). Attitude extremity as one feature of attitude strength (see Krosnick and Petty, 1995), was calculated by computing the deviation of the participants’ responses from neutrality on the feeling thermometer scale (for operationalizing attitude extremity, see Krosnick and Smith, 1994). First, the absolute difference between each participant’s score and the scale mean was calculated. Next, on the new computed item, participants with values from the lowest through the median were selected as low-attitude-strength groups and the rest as the high-attitude-strength group (Hungary_median_: 20.35; Romania_median_: 25.52; Slovakia_median_: 20.42; France_median_: 20.14; Ireland_median_: 22.74). Correlations between the variables, descriptive statistics of all the items, and the items themselves can be found in the Supplementary Material.

### Network Estimation

For each country, a pair of high- versus low-attitude-strength networks were estimated. Using the Extended Bayesian Information Criterion function EBICglasso from the R package qgraph (Epskamp et al., 2012), correlation matrices were inverted into partial correlation matrices to obtain unique statistical associations between all possible pairs of nodes. The correlation matrices were computed through pairwise complete observations to keep all the participants with missing values in the analyses. Also, a regularization technique, LASSO (least absolute shrinkage and selection operator), was employed to control the effects of redundant correlations by setting small coefficients to zero (Friedman et al., 2008).

### Network Comparison

As the main analysis of this study, we compared global connectivity of all high- versus low-attitude-strength networks for each country using the R package “NetworkComparisonTest” (NCT; van Borkulo et al., 2017). We applied a permutation method with 1,000 iterations to examine if high-attitude-strength networks in each country are significantly more connected in comparison with low-attitude networks. In addition, networks were examined as to whether they are structurally different, meaning, for any pair of networks, if there is any edge weight that is significantly different.

### Network Inference

To identify the most influential nodes in high-attitude networks, we computed centrality metrics. Centrality refers to the extent that a node is influential in its interactions with other nodes in a network. Among several centrality metrics, we chose *strength* and *node predictability*. Strength is the sum of all edge weights that a node acquires in relation to all other nodes (Barrat et al., 2004). Using the R package “mgm” (Haslbeck, 2015), we computed the node predictability of each item, which is the proportion of variance for each node explained by all other nodes on average.

### Network Stability

Employing R package bootnet (Epskamp et al., 2018), we computed centrality and edge weight accuracy of all networks. A network is considered stable (i.e., the centrality indices are interpretable) if the order of a centrality index is identical after re-estimating the network with a smaller number of participants, that is, if the correlation stability coefficient (CS-coefficient) is preferably higher than 0.5 and no smaller than 0.25. CS-coefficient is the quantification of the maximum proportion of cases dropped, with 95% probability, so that centrality metrics or edge weights of the remaining cases correlate with those of the original network higher than 0.7 (Epskamp et al., 2018). In addition, bootstrapping with 95% confidence intervals around the edge weights was performed for all networks as an indicator of edge weight accuracy.

## Results

### Network Estimation

**FIGURE 1:**
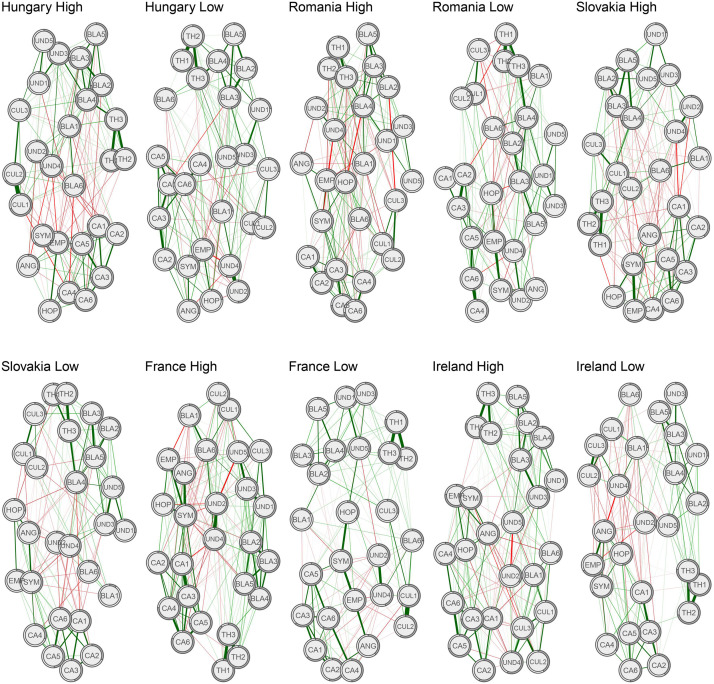
Regularized partial correlation networks of high versus low attitude strength. Node predictability is highlighted by the gray line around each node. Red lines depict negative correlation coefficients, and the thickness of the lines represents the magnitude of partial correlation coefficients. UND1, Undeserved Benefit_1; UND2, Undeserved Benefit_2; UND3, Undeserved Benefit_3; UND4, Undeserved Benefit_4; UND5, Undeserved Benefit_5; CUL1, Cultural Difference_1; CUL2, Cultural Difference_2; CUL3, Cultural Difference_3; BLA1, Blatant Stereotyping_1; BLA2, Blatant Stereotyping_2; BLA3, Blatant Stereotyping_3; BLA4, Blatant Stereotyping_4; BLA5, Blatant Stereotyping_5; BLA6, Blatant Stereotyping_6; CA1, Collective Action_1; CA2, Collective Action_2; CA3, Collective Action_3; CA4, Collective Action_4; CA5, Collective Action_5; CA6, Collective Action_6; EMP, Empathy; SYM, Sympathy; ANG, Anger; HOP, Hope; TH1, Perceived Threat_1; TH2, Perceived Threat_2; TH3, Perceived Threat_3. The green lines represent positive correlations.

Five pairs of high- and low-attitude-strength networks for each sample are depicted in Figure 1. Out of 351 possible edges, networks of high attitude strength were found to have a greater number of non-zero edges (Hungary: 166 vs. 160; Romania: 177 vs. 153; Slovakia: 173 vs. 147; France: 184 vs. 145; Ireland: 173 vs. 145).

### Network Comparison

The global connectivity of every network of high attitude strength was significantly higher compared to that of their corresponding low-attitude-strength network (Hungary: 12.38 vs. 11.64, *p* = 0.03; Romania: 11.85 vs. 10.46, *p* < 0.001; Slovakia: 12.17 vs. 10.97, *p* = 0.005; France 13.48 vs. 11.77, *p* < 0.001; Ireland: 12.94 vs. 11.59, *p* < 0.001). In addition, none of our network pairs showed a significant difference between their edge weights. This implies that high networks did not structurally differ from their corresponding low networks, and the only difference was in their global connectivity.

As mentioned above, to measure attitude extremity, the absolute difference of each participant’s response from the mean value was computed on a feeling thermometer scale. Next, two sub-samples of high and low attitude extremity were created for each country by splitting the datasets by the median of the computed item. As a sensitivity analysis, we split the datasets by 40th–60th as well as 60th–40th percentiles. We ran 10 additional permutation tests. For 8 out of 10 of the comparisons, the effect was still significant. Only in the case of Hungary in the 40th–60th percentile split, we did not find a significant difference, and in the 60th–40th percentile split, the difference was marginally significant (*p* = 0.053).

As another sensitivity analysis, we estimated the networks by a different technique. We binarized all the 27 nodes into zero (from 1 to 4 as not holding the belief) and one (from 5 to 7 as holding the belief) and re-estimated weighted networks with an eLasso technique using the R package IsingFit (van Borkulo and Epskamp, 2015). The eLasso technique regresses all the nodes on all other nodes and regularizes all the regressions controlling for the multicollinearity problem when many variables are regressed on each other (Friedman et al., 2008). Next, the best model fitting the extended Bayesian information criterion is selected (Foygel and Drton, 2010). We then compared all the corresponding high and low networks again by a permutation test with 1,000 iterations. The results were similar to the main analyses, as all of the high-attitude-strength networks showed a significantly higher global connectivity compared to those of low-attitude networks. Moreover, centrality values and network stabilities were also similar to the networks estimated by EBICglasso.

### Network Inference

**FIGURE 2:**
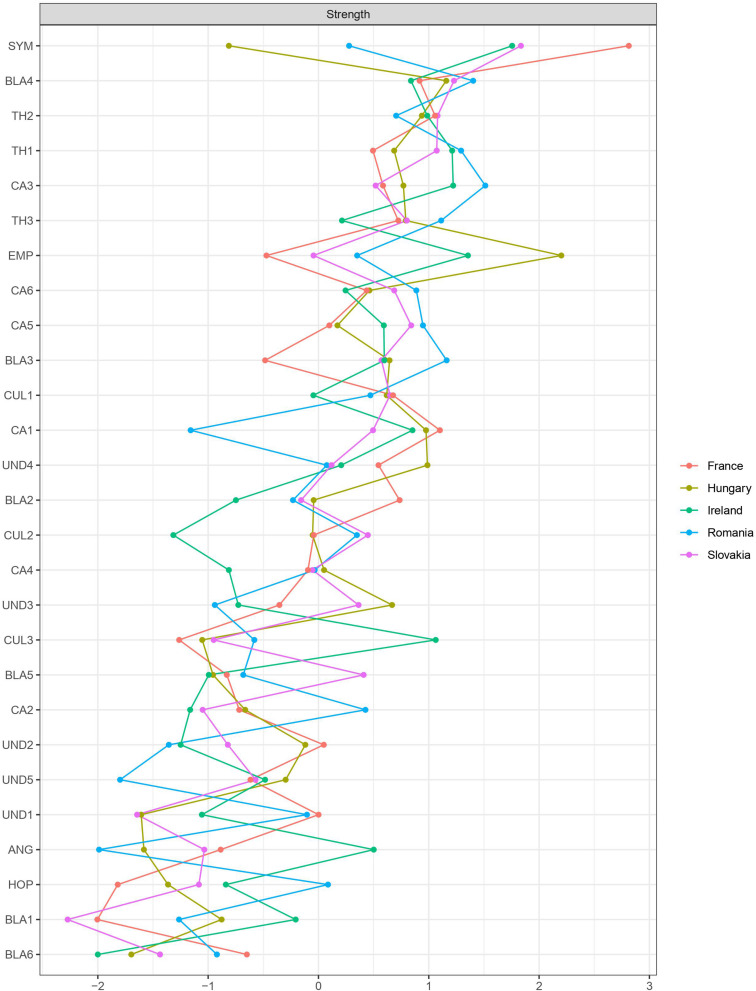
Strength centrality plot of the full-size regularized networks showing standardized *z*-score values of strength centrality. Strength measures the sum of all the regularized partial correlation coefficients for each node.

Figure 2 shows the strength centrality values of all the items of the full-size networks (see the Supplementary Material for further details of the centrality values of all the full-size as well as high- and low-attitude networks). On average, the most central values were found to be empathy in Hungary, perceived threat to national identity in Romania, and sympathy in Slovakia, France, and Ireland. Regarding node predictability, perceived threat to national identity was predicted by other variables to the highest extent in all the full-size networks. Moreover, the order of centrality values of the full-size networks was highly similar to those of the corresponding high- and low-attitude networks.

### Network Stability

Regarding strength centrality, all networks were found to be stable—CS-coefficients were higher than 0.5. Moreover, the edge weights were sufficiently accurate for all networks; the confidence intervals were small enough so that edge weights were interpretable (see Supplementary Material for more details).

### Additional Check

**FIGURE 3:**
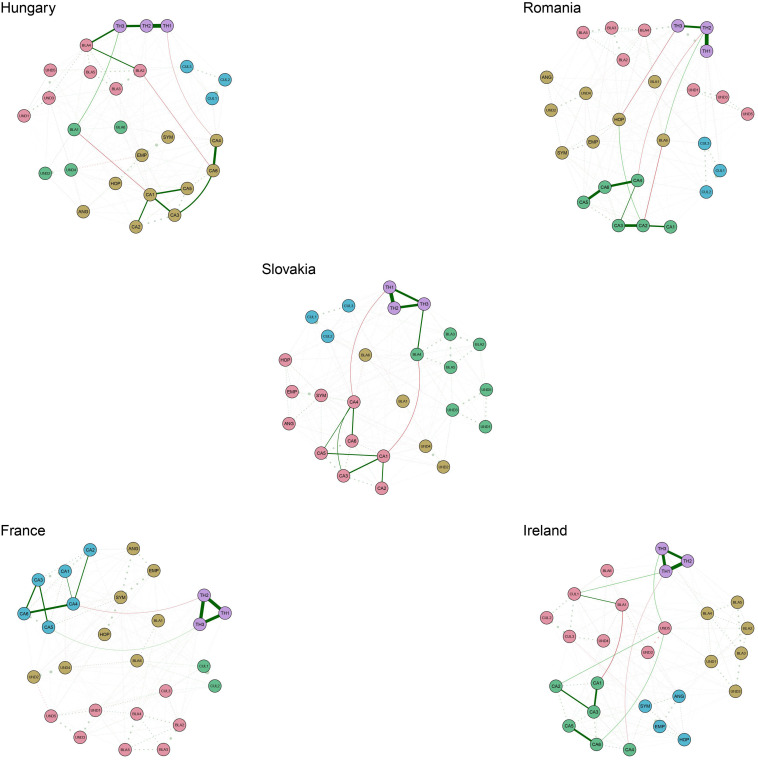
Shortest pathways from perceived threat to national identity to collective action tendencies in the full-size networks. The solid lines depict the edges that belong to the shortest paths. The same colored circles belong to the same community.

As an additional check, we also tested if the structure of anti-Roma bias fits with the intergroup bias structure proposed by Fiske (2015)—social structure predicting stereotypes, which predict emotional prejudice, which in turn predicts behavioral tendencies. We examined the shortest paths from perceived threat to national identity nodes (considered as social structure) to collective action tendency nodes (considered as behavioral tendencies). In all the full-size networks, using the R package EGAnet (Golino and Christensen, 2019), we estimated the number of dimensions, and with the pathways function from the R package qgraph (Epskamp et al., 2012), we examined the shortest paths. Figure 3 shows that there are several shortest paths going from perceived threat to national identity nodes to collective action tendency nodes through the nodes on stereotypical evaluations, while there are also direct paths. However, we do not see the role of emotions in the pathways. The reason should be due to the nature of the intergroup emotions measured in this study, which are prosocial emotions such as hope and empathy as opposed to prejudicial emotions such as contempt and disgust. Overall, the pathways seem to be more or less consistent with the theoretical framework suggested by Fiske (2015). Similar pathway analyses for both high- and low-attitude-strength networks are visualized in the Supplementary Material.

### Discussion

The CAN model (Dalege et al., 2018) was used to examine network connectivity in terms of the evaluative responses on the presidential candidates and found that network connectivity predicts the extent to which individuals are interested in political campaigns. In the current study, we supplemented the connectivity hypothesis by testing it in the context of anti-Roma bias. Using a network approach, we investigated if attitude strength would significantly be associated with stronger connectivity in the networks of a set of stereotypical, emotional, and behavioral reactions toward the Roma people. The findings supported our assumption in all pairs of high versus low networks estimated from the nationally representative samples collected in Hungary, Romania, Slovakia, France, and Ireland. That is, for those who hold a stronger attitude toward the Roma, relevant stereotypical, emotional, and behavioral evaluations are causally interrelated to a significantly higher extent. Moreover, we went beyond the previous research by framing network connectivity as a theoretical justification for future intervention-based research in the context of anti-Roma bias in particular and intergroup relations in a broader scope.

Due to the cross-sectional nature of the study, however, we did not examine the other two empirical findings of the CAN model: the relationship between network connectivity and stability of the attitude in time and its impact on actual behavior. Employing longitudinal designs, future research should consider if this would also be the case with regard to stereotypical evaluations. Moreover, we measured attitude strength by computing the participants’ deviations from neutrality on a feeling thermometer scale. However, extreme responses might not necessarily be due to the strength of the attitude but, rather, the individuals’ response styles. Future research should consider other features and/or measurements and operationalizations of attitude strength. Further, in the current research, we measured behavioral intentions through collective action tendencies; future research could include different measurements such as the preference for contact with Roma people.

Previous research shows that cognitive consistency is a sine qua non factor in configuration of an attitude and the process of its change (e.g., Simon et al., 2004; Monroe and Read, 2008). We also know that the need for cognitive consistency would increase as the attitude strength toward an object increases (see Howe and Krosnick, 2017). By showing that network connectivity is a proxy measurement of attitude strength with regard to anti-Roma evaluations as well, the practical implication of our findings would be to identify and target the most central nodes in anti-Roma attitude networks. This would be a useful means for intervention efforts to combat anti-Roma bias, as in case the most central nodes are at odds with the others, the system should tend to retain consonance, as the connectivity between the nodes is an expression of attitude strength and its related properties such as consistency and stability. This requires further empirical investigations, concerning research on stereotype dynamics, of whether interventions based on the variables with the highest degree of centrality would actually render the most favorable results.

Furthermore, our findings show that regarding the node predictability metric, perceived threat to national identity in all the networks, and regarding the strength metric, empathy in Hungary, perceived threat to national identity in Romania, and sympathy in Slovakia, France, and Ireland were the most central values. Since all the most central values are of an affective nature, our findings suggest that interventions may induce the most favorable impact if the focus were on affective components rather than cognitive components (stereotypes for example) of the social perception of the Roma. This is consistent with the Intergroup Emotions Theory (e.g., Mackie et al., 2008) as well as the Behaviors from Intergroup Affect and Stereotypes (BIAS) Map (Cuddy et al., 2007), which suggest the crucial role of intergroup emotions in predicting relevant behavior. Moreover, our findings also resonate with the literature on intergroup anxiety, proposing the central role of the affective component of intergroup anxiety in prejudice reduction interventions (see Stephan, 2014).

In short, we argue that employing a network approach, by taking network connectivity as a theoretical backbone into consideration, could be a useful tool to depict a complex representation of stereotypical evaluations that have direct and unique connections with each other, to identify values with the strongest associations. Finding the most influential values would enable us to carry out the most effective attitude change interventions. In addition, we propose that the nature and order of central values, as well as other properties of the network dynamic of high-attitude-strength networks, should be taken into account as a perhaps more informative picture for understanding the nature of interconnectedness between different anti-Roma stereotypical evaluations.

## Data Availability Statement

The datasets generated for this study are available on request to the corresponding author.

## Ethics Statement

The studies involving human participants were reviewed and approved by the Eötvös Loránd University, the approval number is 238/2019. The Ethics Committee waived the requirement of written informed consent for participation.

## Author Contributions

HS and MH developed the research question. HS carried out the data analyses and wrote the manuscript. MH, AK, BL, XP, MP, MB, AE-V, CB, YM, AO’C, and AM carried out the data collection and also supervised the research through the critical revision of the manuscript conceptually and analytically. All authors discussed the results and approved the final version of the manuscript to be published.

## Conflict of Interest

The authors declare that the research was conducted in the absence of any commercial or financial relationships that could be construed as a potential conflict of interest.

## Footnotes

1 We improved the original 16-item scale by including reversed items and made the cultural subscale unambiguously about cultural recognition. These revisions were made as part of project PolRom (www.polrom.eu). This paper is the first publication of the new scale.
