# Supplementary material for: Anti-roma Bias (Stereotypes, Prejudice, Behavioral Tendencies): A Network Approach Toward Attitude Strength
Source: Front Psychol. 2020 Sep 30;11:2071. doi: 10.3389/fpsyg.2020.02071 (PMC7554240; doi:10.3389/fpsyg.2020.02071)
Supplement: Supplementary file 4 [file Table_1.DOCX]

Supplementary Material

## Supplementary Figures

**
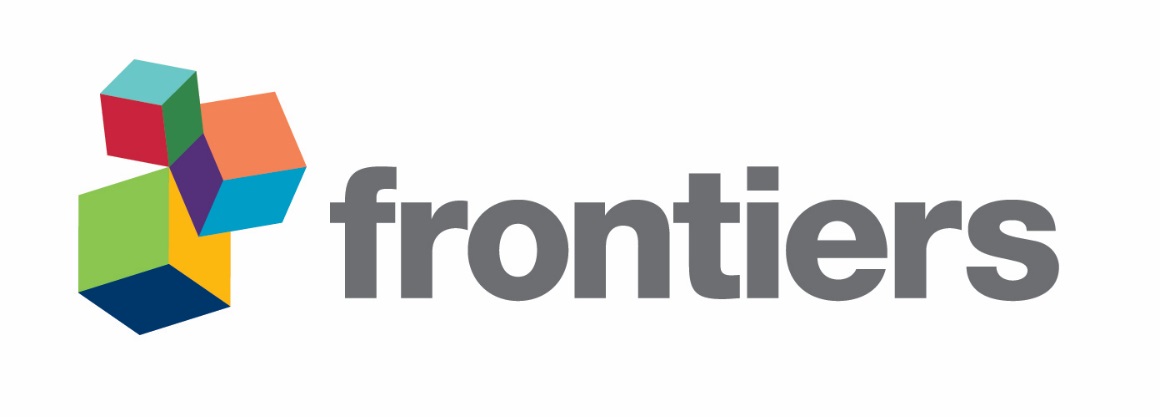
**

**Supplementary Figure 1.** Hungary full-size network (Edge stability)

**Supplementary Figure 1.** Hungary high attitude strength network (Edge stability)

**Supplementary Figure 2.** Hungary low attitude strength network (Edge stability)

**Supplementary Figure 3.** Romania full-size network (Edge stability)

**Supplementary Figure 4.** Romania high attitude strength network (Edge stability)

**Supplementary Figure 5.** Romania low attitude strength network (Edge stability)

**Supplementary Figure 6.** Slovakia full-size network (Edge stability)

**Supplementary Figure 7.** Slovakia high attitude strength network (Edge stability)

**Supplementary Figure 8.** Slovakia low attitude strength network (Edge stability)

**Supplementary Figure 9.** France full-size (Edge stability)

**Supplementary Figure 10.** France high attitude strength network (Edge stability)

**Supplementary Figure 11.** France low attitude strength network (Edge stability)

**Supplementary Figure 12.** Ireland full-size network (Edge stability)

**Supplementary Figure 13.** Ireland high attitude strength network (Edge stability)

**Supplementary Figure 14.** Ireland low attitude strength network (Edge stability)

**Supplementary Figure 15.** Low attitude strength networks (Pathways)

**Supplementary Figure 16.** High attitude strength networks (Pathways)

## Supplementary Tables

**Supplementary Table 1.** Correlations between the variables (Hungary)

**Supplementary Table 2.** Correlations between the variables (Romania)

**Supplementary Table 3.** Correlations between the variables (Slovakia)

**Supplementary Table 4.** Correlations between the variables (France)

**Supplementary Table 5.** Correlations between the variables (Ireland)

**Supplementary Table 6.** Means and standard deviations of all the items

**Supplementary Table 7.** Centrality values of the full-size networks

**Supplementary Table 8.** Centrality values of the low attitude strength networks

**Supplementary Table 9.** Centrality values of the high attitude strength networks

**Supplementary Table 10 and Table 11.** Demographic information of each sample and their corresponding population.

**

## Items

*Undeserved Benefit*

1. The real damage is caused by organizations which offer an undeserved advantage to Roma people.
2. Roma people get given less government money than they should be given (Reversed).
3. Roma people in this country are given preferential treatment in certain aspects.
4. Roma people should be offered more support than they currently receive (Reversed).
5. The only racial discrimination in [country] these days is in favor of Roma people.

*Cultural Difference*

1. The Roma can be proud of their cultural heritage.
2. Roma people have rich artistic traditions.
3. There is nothing special about the cultural heritage of the Roma (Reversed).

*Blatant Stereotyping*

1. Roma people do not make more criminal acts than other people (Reversed).
2. There are very little proper or reasonable Roma people.
3. Roma people do not have a positive relationship to work, they are lazy.
4. The growing Roma population threatens the security of society.
5. Roma people usually have a lot of children, for which they do not give enough care.
6. It is not right that there are still clubs where Roma people are not allowed to enter (Reversed).

*Collective Action Tendencies*

1. I would participate in some form of action (e.g. signing a petition) defending the rights of the Roma.
2. I would publicly express my concern about racism against the Roma by posting on social media or in other ways.
3. I would motivate my friends and acquaintances to participate in actions for the human rights of Roma people.
4. I would donate clothing, school supplies or toys for Roma families.
5. I would do some kind of volunteer work for an organization that helps Roma people.
6. I would motivate others to donate for the Roma.

*Empathy*

I feel empathy with Roma people.

*Sympathy*

I feel sympathy with Roma people.

*Anger*

I feel anger about the treatment of Roma people.

*Hope*

I feel hopeful about the future of Roma people.

*Perceived Threat*

1. Roma people are a threat to [country]’s culture.
2. [country]’s values and traditions are threatened by the presence of Roma people.
3. [country]’s national identity is threatened by the large number of Roma people.
